# Microstructure Evolution in High Purity Aluminum Single Crystal Processed by Equal Channel Angular Pressing (ECAP)

**DOI:** 10.3390/ma10010087

**Published:** 2017-01-22

**Authors:** Jinfang Dong, Qing Dong, Yongbing Dai, Hui Xing, Yanfeng Han, Jianbo Ma, Jiao Zhang, Jun Wang, Baode Sun

**Affiliations:** Shanghai Key Laboratory of Advanced High-temperature Materials and Precision Forming, Shanghai Jiao Tong University, Shanghai 200240, China; jfangdong@hotmail.com (J.D.); dongqing19@163.com (Q.D.); ybdai@sjtu.edu.cn (Y.D.); yfhan@sjtu.edu.cn (Y.H.); kaixnjb@sjtu.edu.cn (J.M.); junwang@sjtu.edu.cn (J.W.); bdsun@sjtu.edu.cn (B.S.)

**Keywords:** equal channel angular pressing (ECAP), high purity aluminum, single crystal, deformation, grain boundary

## Abstract

Aluminum single crystal with 99.999% purity was deformed at room temperature by equal channel angular pressing (ECAP) up to 16 passes. Grain size and misorientation of processed samples were quantitatively characterized by TEM and EBSD. The results show that the refinement efficiency of high purity aluminum single crystal was poor in the initial stage. Extrusion by fewer ECAP passes (*n* ≤ 8) resulted in only elongated grains containing a large number of subgrains and small misorientations between grains. Stable microstructures of nearly equiaxed grains with high misorientations were obtained by 15 passages, indicating that the initial extremely coarse grains and highly uniform grain orientation are not conducive to the accumulation of strain energy. The initial state of high purity aluminum has a significant effect on the refining efficiency of the ECAP process.

## 1. Introduction

High purity aluminum (Al content ≥ 99.999%), as an important raw material in industry, exhibits a much better performance than general pure aluminum. For instance, it can serve as a sputtering target material to prepare thin films used widely in integrated circuits. Both sputtering rate and thin film performance are strongly correlated with the microstructure of target materials, and fine and homogenous grains are generally required for the applications [1]. Traditional methods (e.g., ball milling, refiner addition) are not suitable for the preparation of high purity aluminum due to the purity limitation, while equal channel angular pressing (ECAP) provides an efficient way to overcome the difficulty [2,3]. ECAP is an attractive method of severe plastic deformation for achieving very significant grain refinement to grain size of submicrometer or even nanometer levels [4,5,6,7].

**Figure 1:**
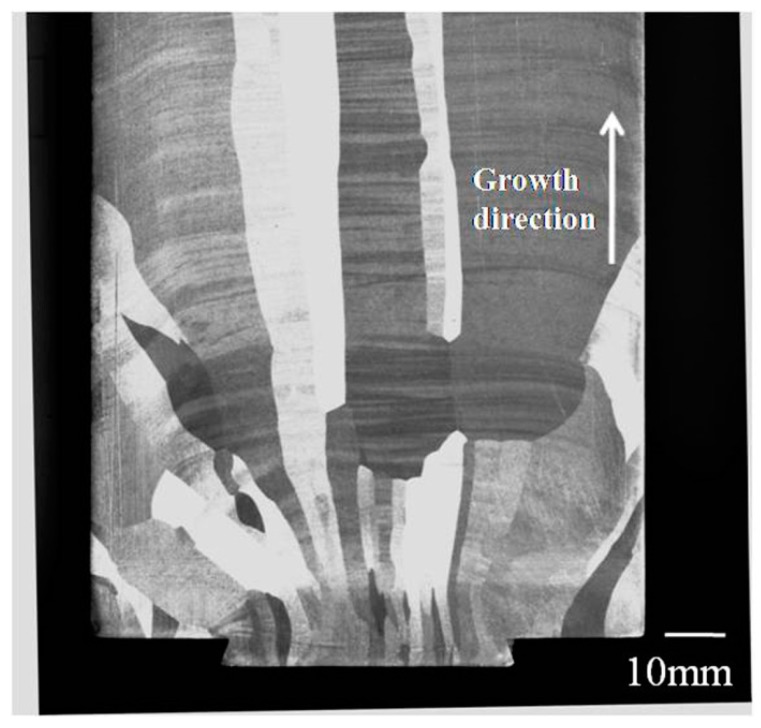
Photo of a cast ingot of high purity (99.999%) aluminum.

To date, a number of studies have been done to investigate the ECAP processes of pure aluminum and high purity aluminum in both experiments and simulations, amongst which some factors such as extrusion route, extrusion passes, and die angles were studied more frequently [8,9,10,11,12,13,14,15]. For general materials, the initial condition of the material is usually neglected due to less obvious particularity. However, it should be noted that in the process of segregation and purification during directional solidification to get 99.999% purity aluminum, it is easy to form a nearly-consistent orientation relationship (growth along [100] direction) and to form coarse columnar grains with diameter up to several centimeters, as shown in Figure 1. These unusual coarse grains can result in substantial decrease of original grain boundaries in cast ingot, and also lead to smaller misorientations among grains. The influence from the above features cannot be neglected in ECAP processes.

In order to study the ECAP refinement process (especially under the conditions of low grain boundary density and small orientation relationship among grains), single crystal is considered as an ideal experimental template. Furukawa et al. applied single crystal, making use of its significant advantage of providing a unique opportunity to select the orientation of the crystal with respect to the pressing direction and the theoretical shear plane to study the fundamental refinement mechanism of ECAP [16,17,18]. Therefore, they only performed an experiment of one-pass extrusion, and did not observe the whole microstructure evolution. In this study, a 99.999% purity aluminum single crystal was applied to deformation by ECAP to high strain (16 passes, equivalent stain of 13.52). Key structural parameters ((sub)grain size and misorientation) during microstructure evolution were quantitatively measured. Based on the evolution features of both parameters, grain refinement and formation process of subgrain boundaries and high angle grain boundaries were analyzed and further discussed without considering the influence from original grain boundaries.

## 2. Experiment

**Figure 2:**
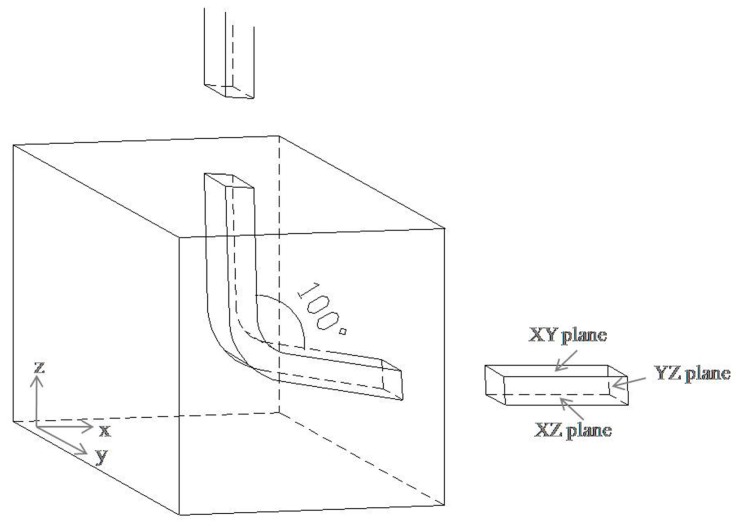
Schematic diagram of equal channel angular pressing (ECAP) die and specimen.

A single crystal rod of high purity (99.999%) aluminum was used in this study. ECAP specimens had square cross-section with dimensions of 10 × 10 mm^2^ and length of 50 mm were cut from the rod using a wire cutting facility. Figure 2 gives a schematic diagram of the ECAP die and specimen. Equivalent strain ε of ECAP is determined by internal angle and external angle of the die per the following relationship [19]:

ε=N/√3(2cot(φ/2+Ψ/2)+Ψcosec(φ/2+Ψ/2)),

where N is the number of ECAP passes.

It can be seen that smaller inner angle leads to larger equivalent strain applied. Furuno used a die with inner angle of 60° for the extrusion of pure aluminum, but dies with acute angle generally need more extruding force, so cracks easily emerge in the sample [20]. It is also difficult to steadily extrude sample to higher stains without any heat treatments during the process, which brings about more difficulty and complexity. Therefore, dies with inner angles above 90° were generally used. While the larger inner angle corresponds to an easier sample extrusion, the larger angle also results in smaller equivalent strain. Moreover, die configuration is correlated with deformation effect. For example, the experimental study for pure Al using a 90° die revealed that route B_C_ was more effective than route C and route A [21]. By contrast, experiments using a 120° die showed that route A was most effective because of negligible stress waste [22]. In order to better observe the microstructure evolution under high strain, route A (no sample rotation between neighboring passes) was selected for sample extrusion. Given the industrial application of high purity aluminum as sputtering target material, a home-designed ECAP die in industrial dimension with inner angle of 100° and outer angle of 30° was successfully applied to obtain a crack-free sample in our previous study [23]. To maintain consistency with our previous study, the same inner angle of 100° and outer angle of 30° was used in this study. The equivalent strain from single pass extrusion is 0.87 according to Equation (1). Before ECAP, mechanical abrading and polishing were used to obtain a mirror-like surface, and Molybdenum disulfide (MoS_2_) was applied to reduce friction. ECAP was carried out at room temperature up to 16 passes at a 18 mm/s extrusion rate. Once the extrusion was complete, the specimen was submerged in water for storage.

After extrusion, the specimens were then slivered into halves along the x axis using a wire cutting facility. To avoid the influences of die friction and contamination on the side wall, the cutting face (*XZ* plane) was selected for further observation. Metallography microscope, NOVA NanoSEM 230 field emission gun scanning electron microscope (FEI Company, Hillsboro, OR, USA), and JEM2100F field emission transmission electron microscope (JEOL Ltd., Tokyo, Japan) were used to observe microstructures and selected area electron diffraction(SAED) patterns. Sampling was conducted within the central area of the *XZ* plane, and more specimen preparation details can be found in our previous study [23].

## 3. Results and Discussion

**Figure 3:**
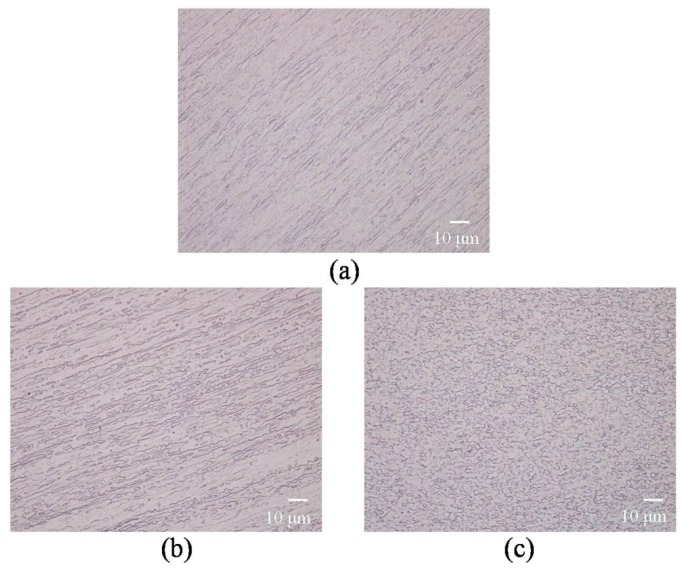
Metallographies of *XZ* plane after different ECAP passes: (**a**) 1 pass; (**b**) 8 passes; (**c**) 16 passes.

The metallographies of 99.999% purity aluminum single crystal after 1, 8, and 16 ECAP passes are shown in Figure 3. After one pass of extrusion, the microstructures show a series of regular parallel sub-crystalline zones elongated along the shear direction, but no grains were clearly observed. Massive long and thin grains were found after eight passes, and a microstructure of homogenous fine nearly equiaxed grains was developed in the entire sample after 16 extrusion passes.

**Figure 4:**
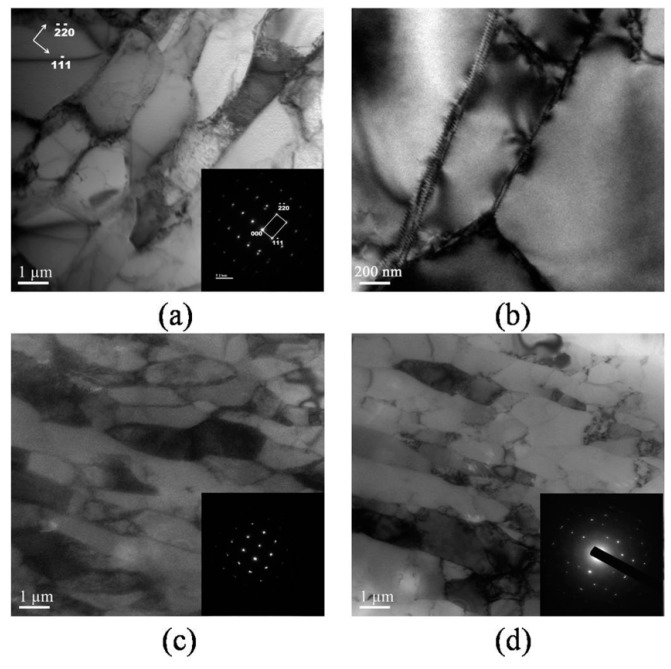
TEM observations of central portion in *XZ* plane after different ECAP passes: (**a**) 1 pass; (**b**) a magnification image after 1 pass; (**c**) 8 passes; (**d**) 16 passes.

The central area of the *XZ* plane was inspected by TEM. The results from 1, 8, and 16 passes are shown in Figure 4. Many small grain structures were observed, and the diameter of a single grain was inhomogeneous after one pass. The SAED patterns (see Figure 4a) show that the crystallographic orientations of small grains are quite small, corresponding to subgrains. Further analysis with SEAD indicated the long axis of the elongated subgrain is along the primary slip system ($\bar{1}$11) [$\bar{1}\bar{1}$0] of aluminum single crystal, and a high density of dislocations was introduced inside the subgrains. As shown in Figure 4b, the high magnification image suggests that the subgrains are almost composed of rearranged dislocations. The SAED patterns after 8 and 16 passes of extrusion indicate that misorientation of grains was enlarged with respect to that after one pass, and some of the boundaries became high angle grain boundaries.

**Figure 5:**
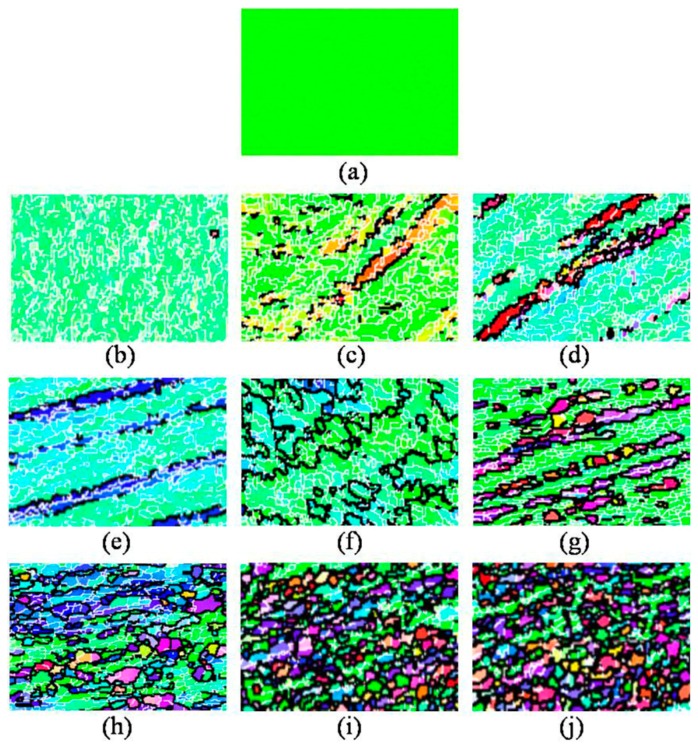
Grain orientation maps after different ECAP passes: (**a**) 0 passes; (**b**) 1 pass; (**c**) 2 passes; (**d**) 4 passes; (**e**) 5 passes; (**f**) 8 passes; (**g**) 12 passes; (**h**) 14 passes; (**i**) 15 passes; (**j**) 16 passes.

**Figure 6:**
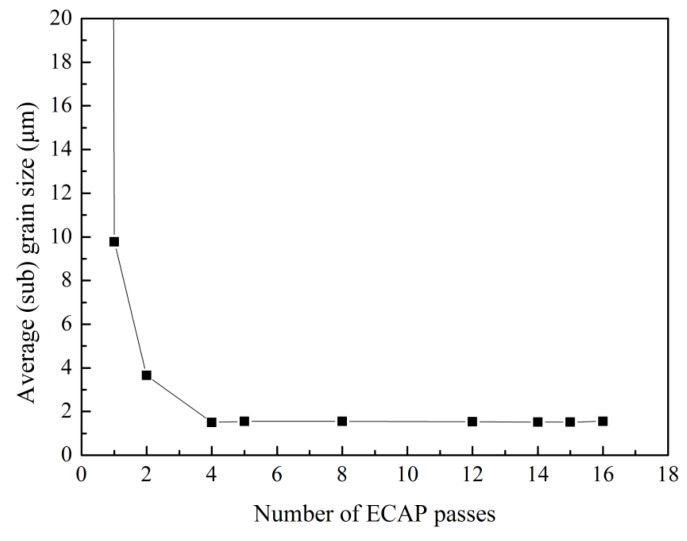
Average (sub)grain size after different numbers of passes.

**Figure 7:**
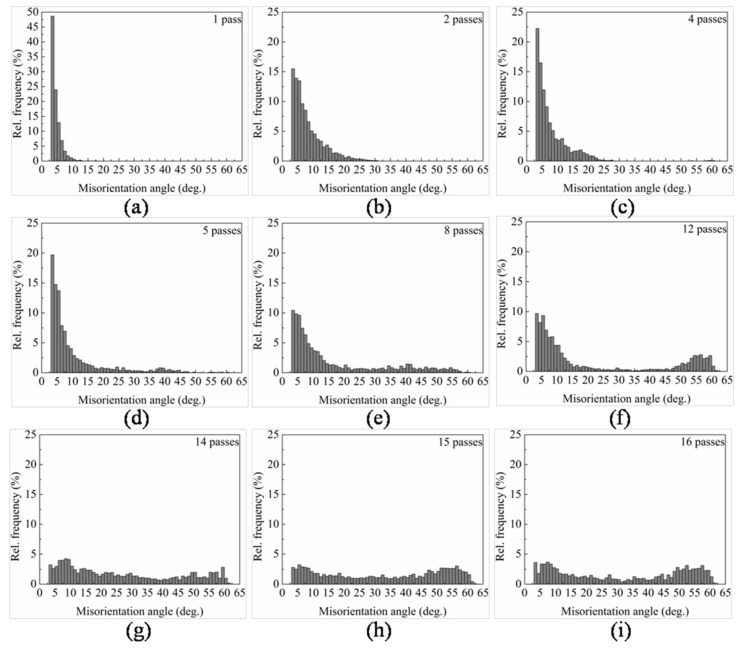
Distribution of boundary misorientation angles after different ECAP passes: (**a**) 1 pass; (**b**) 2 passes; (**c**) 4 passes; (**d**) 5 passes; (**e**) 8 passes; (**f**) 12 passes; (**g**) 14 passes; (**h**) 15 passes; (**i**) 16 passes.

**Figure 8:**
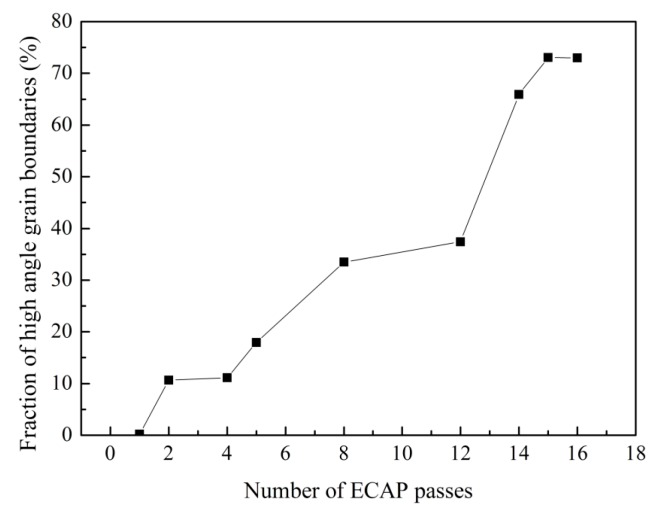
Fraction of high angle grain boundaries after different numbers of passes.

Previous studies have shown that the microstructure evolution during ECAP generally includes two processes: decreasing of grain size and the formation of high angle grain boundaries, and it is known that dynamic recrystallization easily emerges in high purity aluminum. EBSD analysis was carried out to better investigate the microstructure evolution with different numbers of ECAP passes. Figure 5b–j show the grain orientation maps of the central area on the *XZ* plane after 1, 2, 4, 5, 8, 12, 14, 15, and 16 passes of ECAP, respectively. As a reference, unprocessed sample is also shown in Figure 5a. Figure 6 compares the average (sub)grain sizes subject to different passes. The distribution of misorientations and the fraction of high angle grain boundaries with different passes are shown in Figure 7a–i and Figure 8, respectively. Clear microstructure evolution can be seen under different strains during the extrusion process. The original 10 × 10 × 50 mm^3^ single crystal can be regarded as an infinitely large grain size. After one ECAP pass, the matrix became composed of elongated subgrains surrounded by thin white lines, where the average (sub)grain size was 9.8 μm and all misorientations were less than 15°. These subgrains are mainly composed of massive rearranged dislocations induced by plastic deformation, as shown in Figure 4b [24]. With further deformation up to four passes, high angle grain boundaries over 15° increased (bold black line in Figure 5c,d) formed by adsorbing and recombining mobile dislocations by already existed subgrains [25]. It is believed that the dislocation accumulation process (intragranular dislocation activities) is able to produce misorientations primarily up to 15°–30° [26]. As shown in Figure 5, misorientations are always below 30° up to four passes. At higher strains, dislocation activities within grains are limited by intergranular strains, which need to be accommodated by subgrain rotation [27]. Misorientations larger than 30° start to appear following the fifth pass. It should be noted that between two and eight passes, high angle grain boundaries always arrange along the shear direction so that a large range of elongated grains first form in the matrix. The main reasons for this microstructure are as follows: the formation of a large area of subgrains with negligible misorientation after extrusion because of the lack of interruption from the original grain boundary in the single crystal, and the continuous deformation along the same shear direction because of the adoption of deformation route A (no sample rotation between neighboring passes). With the accumulation of the deformation strain, dynamic recrystallization becomes more intense. From the eighth pass, fine grain and the fraction of high angle grain boundaries increase dramatically until the fifteenth pass (equivalent strain of 13.05), where homogenous nearly equiaxed grains form across the whole matrix and the fraction of high angle grain boundaries reaches 73%. Microstructure was almost unchanged with one more pass. It depends on a balance between dislocations generated from plastic deformation and the dislocations adsorption by grain boundaries [28]. Small grains also attain stable positions by intercoordination and rotation between each other, and finally form the equilibrium recrystallized microstructure.

According to some previous studies about then ECAP process of fine polycrystalline material, the equilibrium state can be obtained by only eight passes (equivalent strain of 8.0), and further deformation to 12–16 passes cannot induce more changes in the microstructure [29,30,31]. Therefore, in comparison with polycrystalline materials, due to the lack of interruption on the activity of the dislocations from original grain boundary, the refinement efficiency of a single crystal in the earlier stage is poor, and 15 passes (equivalent strain of 13.05) are necessary to reach the equilibrium state of the microstructure. Therefore, it is believed that the extremely coarse initial grains and highly uniform grain orientation are not conducive to the accumulation of strain energy. The initial state of high purity aluminum ingot has a significant effect on the refining efficiency of ECAP process. In the future, we will further investigate the influence of different routes on the refinement efficiency of single crystal.

## 4. Conclusions

High purity aluminum single crystal was extruded up to 16 passes by route A at room temperature to study the ECAP process under the initial condition with both extremely low density of grain boundaries and small misorientations of grains. It was found that for high purity aluminum single crystal, extrusion by fewer ECAP passes (*n* ≤ 8) results in only elongated grains containing a large number of subgrains and small misorientations between grains. By further increasing the number of passes (up to 15 passes), stable microstructures of nearly equiaxed grains with large misorientations were obtained. The results show that with respect to single crystalline material, the lack of high angle grain boundary in the initial state leads to a poor refinement efficiency in the earlier stage. This indicates that the extremely coarse initial grains and highly uniform grain orientation are not conducive to the accumulation of strain energy. The initial state of high purity aluminum ingot has a significant effect on the refining efficiency of the ECAP process. In the future, we will further investigate the influence of different routes on the refinement efficiency of single crystal.
